# Universal Health Coverage (UHC) in Iran

**Published:** 2018-07

**Authors:** Moggan LETAFAT, Tina BEYRANVAND, Aidin ARYANKHESAL, Meysam BEHZADIFAR, Masoud BEHZADIFAR

**Affiliations:** 1. Dept. of Health Services Management, School of Health Management and Information Sciences, Iran University of Medical Sciences, Tehran, Iran; 2. Dept. of Epidemiology, Faculty of Health and Nutrition, Lorestan University of Medical Sciences, Khorramabad, Iran; 3. Health Management and Economics Research Center, Iran University of Medical Sciences, Tehran, Iran

## Dear Editor-in-Chief

Universal Health Coverage (UHC) is one of necessities of achieving optimal health and equity in any nation (1). Hence, most leading health systems make their best effort to include maximum mixture of care in their package, increase access to high quality services and increase the coverage of population (2). Such access is necessary to be achieved in all aspects of health, including prevention, treatment, rehabilitation and relief of pains across the suffering patients. UHC counts as a key factor, internationally, therefore, WHO, considers it in its Millennium Development Goals (MDG) as the third goal (3). WHO defines the UHC as ability to access all community health services by people who need them along with provision of financial support by the governments. UHC can result in higher the level of health equity in populations. Nevertheless, UHC’s definition in any country is associated with its economic, social, political, and cultural status. The UHC cube, introduced by the WHO, has three axes; direct costs (proportion of the cost covered), services (services covered), and population (covered). All three axes should be promoted parallel, otherwise, disproportionate growth across axes can lead to reverse results and reduction of equity (4, 5). Iran, same as many other countries, has made certain efforts to achieve UHC. In this post, we aimed to examine briefly Iran’s status in any axes of the UHC cube in 2016.

Based on World Bank’s report, the amount paid out of patients’ pocket between 1995 and 2014, dropped from 80.5% to 59.5% (6). The amount of this indicator based on an official report published by Iran’s Ministry of Health and Medical Education (MoHME) was 40% in 2016 (7). Such decrease shows Iran’s will to improve the situation, although there are very much to do compare to developed nations’ situation. The goal of this indicator is to increase the number and diversity of services provided to patients, especially the poor ones. Iran’s Health Network System was established in 1986 in order to increase access to primary health care in remote parts of the country. Then in 2006, expansion of the insurance program for the villagers, along with the establishment of family physician and the referral system in all villages and cities with a population of fewer than twenty thousand people, increased access to first-level service packages. Currently, the basic service package in the form of family physician plan is accessible free of charge for people in villages and cities with a population of fewer than twenty thousand populations. The second and third level services are provided by public and private hospitals around the country. There are still some services, such as the heart, liver, bone marrow transplant, plastic surgery, joint replacement that are not covered by insurance companies. Nevertheless, in recent years the list of covered services has increased to include more services related to the diagnostic-therapeutic procedures, infertility, as well as some dental related services. The MoHME has faced challenges with defining basic package of services covered by insurance companies due to multiplicity of the insurance funds in the country, the limitation of financial resources and lack of attention to cost management, lack of the using evidence-based medicine for effective interventions and lack of proper policies to create harmony between the insurance companies and the MoHME. The implementation of the free coverage for the people without insurance through Iran’s Health System Transformation Plan increased the coverage percent to more than 95% (7).

**Fig. 1:**
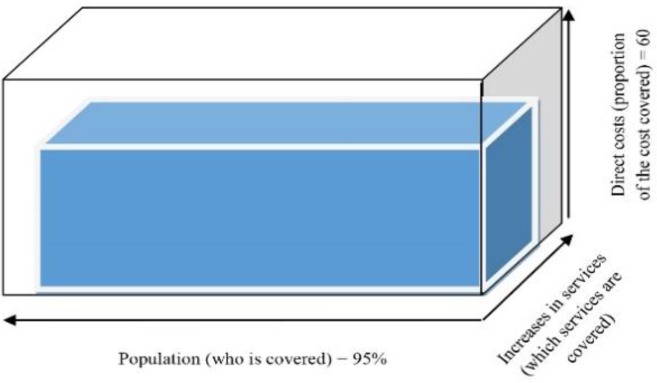
The three axes UHC cube in Iran

The UHC cube in Iran has enlarged through all its three axes and got closer to WHO’s desired cube which indicates improved level of health equity in Iran (8). The most important problems against achieving the UHC are, usually, inadequate budgets, lack of clear borders between public and private systems, multiplicity of insurance organizations and insurance funds, drastic changes in epidemiology of diseases and demographic characteristics, lack of accountability to demands of society due to the limitations of manpower employed in health sector and negligence of social variables in this sense (9, 10). The three axes UHC cube in Iran showed in Fig. 1.

Hence, reaching the ideal cube axes needs more political commitment from the government part, a change of attitude to the health sector and making furthermore efforts for reaching an efficient health system.
